# Circulating Serum MiRNA-8074 as a Novel Prognostic Biomarker for Multiple Myeloma

**DOI:** 10.3390/cells11040752

**Published:** 2022-02-21

**Authors:** Aneta Szudy-Szczyrek, Radosław Mlak, Michał Mielnik, Marcin Mazurek, Sylwia Chocholska, Martyna Podgajna, Michał Szczyrek, Iwona Homa-Mlak, Teresa Małecka-Massalska, Marek Hus

**Affiliations:** 1 Chair and Department of Haematooncology and Bone Marrow Transplantation, Medical University of Lublin, 20-081 Lublin, Poland; michal.mielnik@umlub.pl (M.M.); sylwiachocholska@umlub.pl (S.C.); marpodgajna@gmail.com (M.P.); 2 Department of Human Physiology, Medical University of Lublin, 20-080 Lublin, Poland; radoslaw.mlak@umlub.pl (R.M.); marcin.mazurek1@umlub.pl (M.M.); iwona.homa@umlub.pl (I.H.-M.); teresa.malecka-massalska@umlub.pl (T.M.-M.); 3 Chair and Department of Pneumonology, Oncology and Allergology, Medical University of Lublin, 20-950 Lublin, Poland; michal.szczyrek@umlub.pl

**Keywords:** microRNA, miRNA-8074, multiple myeloma, prognostic factor, predictive factor, bortezomib, thalidomide, epigenetics

## Abstract

MiRNA-8074 is a molecule with the potential to regulate the expression of key genes related to the pathogenesis of multiple myeloma (MM), i.e., *TP53*, *MYC*, *MAPK1*, and *KIAA*. We analyzed the predictive and prognostic value of miRNA-8074 expression in MM patients. In total, 105 newly diagnosed MM patients treated with thalidomide (n = 27), bortezomib (n = 41) and bortezomib with thalidomide (n = 37) were studied. For miRNA analysis, the column method and the Real-Time PCR technique with specific TaqMan Fast Advanced Master Mix and TaqMan probes were used. Factors that were associated with a significant reduction in progression-free survival (PFS) included: ECOG > 1, ISS stage III, low hemoglobin, thrombocytopenia, hypoalbuminemia, abnormal renal function, elevated creatinine, GFR < 60 mL/min/1.73 m^2^, elevated LDH, del(17p), t(11;14), the use of a single drug regimen (thalidomide or bortezomib) and high miRNA-8074 expression (HR = 2.01, 95% CI: 1.16–3.49; *p* = 0.0233). In addition to the known prognostic factors, such as ECOG > 1, Durie–Salmon stage III, diagnosis of light chain disease or non-secreting MM, renal failure, hypoalbuminemia, hypercalcemia, high β2-microglobulin, elevated LDH, and t(14;16), a high expression of miRNA-8074 was significantly associated with a higher risk of death (HR = 4.12, 95% CI: 2.20–7.70; *p* = 0.0009). In summary, miRNA-8074 may be a useful diagnostic tool to assess the prognosis in MM patients.

## 1. Introduction

Multiple myeloma (MM) is a hematological malignancy characterized by the clonal proliferation of plasma cells in the bone marrow. The worldwide incidence of MM is approximately 160,000 cases per year, and the mean age of onset is 70 years old. In its advanced form, the disease leads to pathological bone fractures and bone marrow and multi-organ failure [1,2]. Despite the huge progress associated with the introduction of proteasome inhibitors (PIs), immunomodulatory drugs (IMiDs) and monoclonal antibodies, MM still remains an incurable disease. Even in patients with complete remission, relapse is very common, with a median survival of approximately 6 years [3,4]. The varied clinical course of MM, differences in the efficacy of specific therapeutic strategies, and different times of chemoresistance development imply the need to identify risk stratification factors that would enable the personalization of therapy and improvement of treatment outcomes.

MicroRNAs (miRNAs) are short (about 22 nucleotides in length) non-coding RNA fragments, which have the ability to regulate gene expression at the post-transcriptional level. Mature miRNAs are selectively bound together with the Argonaute AGO2 protein with endonucleolytic activity to translation silencing complexes (RNA-induced silencing complex, miRISC). These complexes have the ability to bind to the 3′UTR regions (untranslated regions) of the mRNA of the target gene, and due to full nucleotide complementarity, they can lead to transcript degradation. In most cases, however, the miRNAs do not show complete complementarity to the 3′UTR and retain the ability to inhibit the translation of the target mRNA [5,6].

A single miRNA can modulate thousands of genes [7]. Genes for miRNAs are often located at or near fragile sites of chromosomes [8]. A common change in gene expression for miRNAs is observed in neoplastic cells, and can be caused by deletions, translocations or amplifications. These mechanisms lead to changes in the expression of target genes. Depending on which genes they influence, miRNAs can function as oncomir-pro-carcinogenic or as suppressor-inhibiting oncogenes. Thus, miRNAs can serve as promising biomarkers for the detection and prognosis of cancer [9,10,11].

The observations to date indicate that disturbances in miRNA expression are closely related to MM pathogenesis, tumor initiation and tumor progression [12,13,14], and miRNAs seem to be an attractive research area for new therapeutic approaches against MM. miRNA-8074 is located on the long arm of chromosome 19 (location 19q13.41). The molecule may influence the expression level of key genes related to MM pathogenesis, i.e., *TP53*, *MYC*, *MAPK1*, *MAF*, and *KIAA* [15]. The aim of our study was to assess the impact of the expression level of circulating miRNA-8074 on the treatment outcomes and survival of patients with MM.

## 2. Material and Methods

### 2.1. Patients

We retrospectively analyzed 105 patients (53 women, 52 men) with newly diagnosed MM, aged 37–87 years (median age 66). Patients were recruited in the Department of Hematooncology and Bone Marrow Transplantation in Lublin from 2015 to 2019. MM diagnosis was based on SLiM CRAB criteria according to the International Myeloma Working Group (IMWG) recommendations. Disease staging was determined according to Durie–Salmon and ISS staging systems. Patients’ performance statuses were evaluated using the ECOG-WHO scale. Abnormal kidney function was defined as a serum creatinine level of higher than 2 mg/dL (176 µmol/L), according to Durie–Salmon substage B [16,17,18].

Patients received thalidomide and/or bortezomib combined with steroids and/or cyclophosphamide as the first-line treatment. Therapeutic regimens included CTD (28-day regimen—i.e., 100 mg of thalidomide daily in combination with 300–500 mg/week of cyclophosphamide, and 10–20 mg/day of dexamethasone (n = 27)), V(C)D (i.e., 1.3 mg/m^2^ of bortezomib i.v. on days 1, 4, 8 and 11 with or without 300–500 mg/week of cyclophosphamide and 10–20 mg/day of dexamethasone on days 1–4 and 8–11 (n = 41)) and VTD (i.e., 1.3 mg/m^2^ of bortezomib i.v. on days 1, 4, 8 and 11 in combination with 100 mg of thalidomide daily and 10–20 mg/day of dexamethasone on days 1–4 and 8–11) (n = 37)).

Treatment was carried out in accordance with the guidelines in force at that time. The choice of chemotherapy was based on patient characteristics, toxicity, comorbidities and drug availability (not all drugs were reimbursed by the National Health Fund). The inclusion criteria were: age ≥ 18, diagnosis of symptomatic MM according to IMWG criteria, and a life expectancy greater than 3 months. For high-risk transplant-eligible patients, bortezomib in combination with thalidomide plus dexamethasone (VTD) was preferred. Bortezomib-based V(C)D regimens were administered to patients with renal impairment. 

Treatment response was assessed according to the current IMWG guidelines and classified as stringent complete remission (sCR), complete response (CR), very good partial response (VGPR), partial response (PR), minimal response (MR), stable disease (SD), or progressive disease (PD), as described elsewhere [19]. The severity of adverse events was assessed according to the Common Terminology Criteria for Adverse Events (CTCAE) v5.0 scale [20]. 

The detailed patient characteristics are presented in Table 1.

This study was conducted in accordance with the principles of the Helsinki Declaration and the International Conference on Harmonisation Good Clinical Practice guidelines. Local ethical approval was obtained from the Bioethical Committee at the Medical University of Lublin (consent number: KE-0254/26/2015). All patients gave written informed consent.

### 2.2. Serum Collection

The studied material was approximately 3 mL of venous blood collected before the treatment into serum gel tubes. The samples were centrifuged at 3000 rpm for 10 min, and serum samples were subsequently pipetted into tubes and stored at −80 °C until further analysis.

### 2.3. MiRNA Analysis in Blood Serum

miRNA isolation from 200 µL of serum samples was performed using the column method with a dedicated kit according to the manufacturer’s recommendations (miRNeasy Serum/Plasma Kit, Quiagen, Toronto, Canada). The Real-Time Polymerase Chain Reaction (Real-Time PCR) technique was performed on a StepOnePlus device (Applied Biosystems, Foster City, CA, USA) according to the manufacturer’s protocol using TaqMan Fast Advanced Master Mix and TaqMan probes (20×) (Assay name: hsa-miR-8074, Assay ID: 480616, Thermo Fisher Scientific, Waltham, USA). The total reaction volume was 20 μL per well. The reaction conditions were enzyme activation at 95 °C, denaturation at 95 °C (40 cycles) and elongation at 60 °C (20 s). The level of miR-8074 expression was normalized to hsa-miR-26a-5p (Assay ID: 47995) as a housekeeping gene using the 2^−ΔCt^ and 2^−ΔΔCt^ formulas. 

### 2.4. Plasma Cell Isolation

Bone marrow mononuclear cells (BMMC) were separated from 3 mL of bone marrow (diluted 10×) by density gradient centrifugation (Biocoll, AG Biochrom, Berlin, Germany). After washing and the assessment of viability, the cells were subjected to separation. Cell separation, after incubation with CD138 antibody, was performed according to the manufacturer’s instructions with the use of the method of magnetic-activated cell sorting (MACS^®^ Cell Separation, Bergisch Gladback, Germany). Subsequently, an assessment of the viability and quantity of plasma cells was performed.

### 2.5. MiRNA and Gene Expression in Bone Marrow Plasma Cells

Total RNA was isolated from separated plasma cells (a maximum of 10^7^ cells were used) with the use of the RNeasy Mini Kit (Qiagen, Canada). miR-8074 expression assessment was performed as described in Section 2.3. The expression of *TP53* and *MYC* genes was assessed with the use of the StepOnePlus device and dedicated kits: High-Capacity cDNA Reverse Transcription Kit, TaqMan™ Fast Advanced Master Mix and TaqMan probes (Thermo Fisher Scientific, Waltham, USA). For miR-8074 analysis, hsa-miR-26a-5p (Assay ID: 47995) as a housekeeping gene was used, whereas for the *TP53* (Assay ID: hs01034249) and *MYC* (Assay ID: hs00153408) genes, *ACTB* (Assay ID: hs99999903) was used as a reference gene. The relative expression of the studied molecular targets was calculated using the 2^−ΔCt^ and 2^−ΔΔCt^ formulas. 

### 2.6. Cytogenetic Assessment

Bone marrow samples of MM patients were used to perform cytogenetic analysis. Abnormalities, such as *TP53* gene deletion, *IGH* gene rearrangements and *CKS1B* gene amplification, were detected through the simultaneous staining of cytoplasmic immunoglobulin with the fluorescence in situ hybridization (cIg-FISH) technique according to the recommendations of Ross et al., with some modifications [21]. The following probes, all from Abbott Molecular (Abbott Park, IL, USA), were used: Vysis TP53/CEP 17 FISH Probe Kit for the detection of del(17p13.1), Vysis IGH/FGFR3 Dual Colour, Dual Fusion Translocation Probe for the detection of t(4;14)(p16;q32), Vysis IGH/MYC/MYC Tri-color, Dual Fusion Translocation Probe for the detection of t(8;14)(q24;q32), Vysis IGH/CCND1 Dual Colour, Dual Fusion Translocation Probe for the detection of t(11;14)(q13;q32), Vysis IGH/MAF Dual Colour, Dual Fusion Translocation Probe for the detection of t(14;16)(q32;q23) and Vysis 1q21 CKS1B SpectrumOrange/1p32 CDKN2C SpectrumGreen FISH Probe Kit for the detection of amp(1q32). Fluorescent microscopic analysis was performed by scoring 100 AMCA-positive plasma cells to determine the frequency of each aberration. The cut-off levels were 20% for deletion/amplification probes and 10% for dual-fusion probes, according to the recommendations of the European Myeloma Network [22,23].

### 2.7. Statistical Analysis 

Statistical analysis was performed using MedCalc software (v.15.8). Because miRNA-8074 expression data were not normally distributed (according to the D’Agostino–Pearson test), we used the non-parametric U-Mann–Whitney test (for comparisons of two groups) and Kruskal–Wallis test (for comparison of more than two groups), and the results are shown as medians (measures of central tendency). The non-parametric Spearman correlation test was performed. Receiver operating characteristics (ROC) curves and area under the ROC curve (AUC) were used to assess the usefulness of miRNA-8074 expression in differentiating patients with the presence or absence of response to treatment. The Kaplan–Meier method and Cox regression were used to assess the probability of disease progression and survival. In the cases of both the composite (e.g., ISS) and its separately constituent variables (e.g., albumin, B2M, etc.), which were statistically significant in the univariate analysis, we only included the first of them in multivariate models. Progression-free survival (PFS) was defined as the length of time from the start of the therapy until disease progression (complete data) or last documented follow-up (censored data), while overall survival (OS) was defined as the length of time from the start of therapy until the patient’s death (complete data) or last documented (censored) follow-up.

## 3. Results

Bone marrow cytogenetic tests for del(13q), del(17p), t(4;14), t(11;14), t(14;16), and t(14;20) were performed in 85 patients. The following molecular aberrations were detected: 17p13.1 deletion (*TP53 locus*) (n = 11, 12.9%), t(4:14) *FGFR3/IGH* (n = 10, 11.8%), t(14;16) *MAF/IGH* (n = 1, 1.2%), t(11;14) *CCND1/IGH* (n = 9, 10.6%), and other less frequent rearrangements with the *IGH* gene (n = 13, 15.3%). Paired bone marrow samples for gene expression analysis were available for 23 patients.

### 3.1. Comparison of the MiR-8074 Expression Depending on Demographic, Clinical and Molecular Factors

We did not observe statistically significant differences in the level of miRNA80-74 expression considering demographic, clinical and molecular variables, which are presented in Table S1.

### 3.2. Correlation between Serum MiRNA-8074 and Selected Demographic, Clinical and Molecular Variables

We noted a statistically significant positive correlation (rho = 0.549) between miRNA-8074, which was assessed in blood serum and bone marrow plasma cells. Moreover, in plasma cells, a significant, positive correlation (rho = 0.476) between the expression of miRNA-8074 and the *MYC* gene was found. On the other hand, the studied miRNA correlated negatively (rho = −0.317) with the expression of the *TP53* gene; however, the result was not statistically significant (Figure S1). We found no statistically significant correlation between the tested miRNA-8074 expression and any of the selected demographic and clinical variables. Detailed data are presented in Table S2.

### 3.3. Correlation between MiRNA-8074 Expression and Chemotherapy Response

We did not find statistically significant differences in the level of serum miRNA-8074 expression depending on the treatment response after 2, 4, 6 or 8 treatment cycles. We also did not fInd a statistically significant usefulness of the studied miRNA in differentiating patients in terms of response to chemotherapy (CTH) after 2, 4, 6 or 8 cycles. Detailed data on the comparison of the miRNA-8074 level and the diagnostic value of this marker in the context of the response to CTH are presented in Table S3.

### 3.4. Outcomes: ProgressionFree Survival

#### 3.4.1. Univariate Analysis

**Figure 1:**
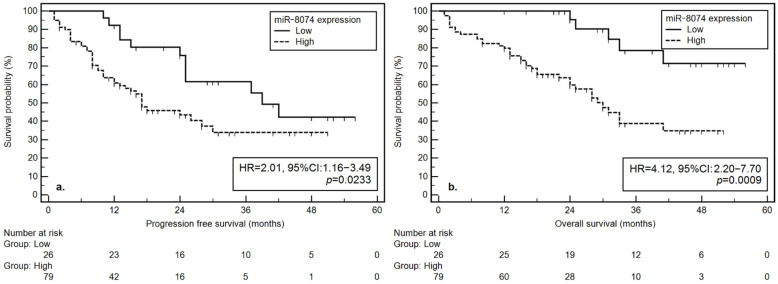
Kaplan-Meier curves from the analysis of miRNA-8074’s influence on (**a**) progression-free survival and (**b**) overall survival.

Based on a univariate analysis, we confirmed the influence of a number of prognostic factors on progression-free survival (PFS). The significant reduction in PFS was related to: a higher disease stage according to the ISS classification (3; HR = 1.82), the presence of a 17p deletion (HR = 2.08), worse renal function (B; HR = 2.31), higher degree of chronic kidney disease (G31-G5D; HR = 2.50), poor performance status (PS: 2–4; HR = 1.73), use of a therapy based only on thalidomide or bortezomib (instead of a combination of the two drugs; HR = 2.64), low hemoglobin (HR = 2.42), low platelet count (HR = 1.81), low albumin (HR = 2.57), elevated LDH (HR = 2.58), elevated creatinine (HR = 2.54), low eGFR (HR = 2.08), presence of del(17p) (2.08), and the presence of t(11;14) *CCND1/IGH* (HR = 2.94). In addition, we observed that a high miRNA-8074 expression was associated with a risk of PFS shortening (17 vs. 39 months; HR = 2.01) (Figure 1).

#### 3.4.2. Multivariate Analysis

A multivariate analysis confirmed the independent prognostic value of PFS reduction for stage 3 disease according to ISS classification (HR = 2.59), the use of therapy based on thalidomide or bortezomib only (instead of a combination of these two drugs; HR = 2.75), thrombocytopenia (HR = 2.54), low albumins (HR = 2.35), LDH (HR = 3.28), and t(11; 14) *CCND1/IGH* translocation (HR = 5.66). The prognostic value of a high expression of the miRNA-8074 for PFS shortening (HR = 2.28) was also confirmed.

Detailed data on PFS are presented in Table 2.

### 3.5. Overall Survival

#### 3.5.1. Univariate Analysis

Based on the univariate analysis, we confirmed the impact of a number of prognostic factors on overall survival (OS). A significant shortening of the OS was associated with: the diagnosis of light chain disease or non-secreting MM (HR = 3.01), a higher disease stage according to the Durie–Salmon classification (III; HR = 3.06), worse kidney function (B; HR = 2.03), poor performance status (PS: 2–4; HR = 2.64), low albumin (HR = 2.29), high LDH (HR = 3.01), high calcium (HR = 1.88), high β2-microglobulin (B2M) level (HR = 3.12), and *IGH/MAF* fusion (HR = 21.92). In addition, we observed that high miRNA-8074 expression was associated with a higher risk of OS shortening (30 months vs. NR; HR = 4.12) (Figure 1).

#### 3.5.2. Multivariate Analysis

The multivariate analysis confirmed the prognostic value in terms of OS shortening for stage III disease according to the Durie–Salmon classification (HR = 4.84), high LDH level (HR = 5.94), and t(14;16) *MAF/IGH* (HR = 81.76). Moreover, we observed that a high expression of miRNA-8074 was associated with an independent, higher risk of OS shortening (HR = 3.97).

Detailed data on the survival analysis are presented in Table 2.

## 4. Discussion

A comprehensive assessment of a number of clinical factors and laboratory results is necessary to assess the prognosis of MM patients. Durie and Salmon’s (1975) classification of MM staging, which assesses tumor mass based on the concentration of hemoglobin, calcium, serum monoclonal protein and osteolytic changes in bone [17], has increasingly been replaced by the International Staging System (ISS) introduced in 2003 [18], and now by the modified International Staging System (R-ISS) [24]. The R-ISS scale takes into account the concentration of B2M, LDH and serum albumin as well as the assessment of cytogenetic risk [24]. Fluorescence in situ hybridization (FISH) is used to analyze cytogenetic changes in neoplastic plasma cells. High-risk aberrations include t(4;14), t(14;16), and t(14;20) translocations, chromosome 17p deletion (del(17p)), hypodiploidy and 1q21 amplification. It is estimated that about 20% of MM patients carry at least one of the above-mentioned aberrations [25,26]. The prognostic tools used in current clinical practice seem to be insufficient to capture the complexity and measure the aggressiveness of MM. With the emergence of new treatment strategies, for them to be effective it is necessary to properly match them to the molecular profile of the tumor. There is an ongoing search for new biomarkers that would be more accurate and thus contribute to a more effective therapy. In general, a prognostic biomarker gives information about the clinical outcome, regardless of therapy, while a predictive biomarker provides information about the response to a particular therapy. Likewise, a molecule that is a predictive biomarker can be a target for therapy [27,28]. An ideal biomarker should be easily accessible in non-invasive procedures, inexpensive to measure, and very specific and sensitive in the diagnosis of the disease.

Endogenous miRNAs influence a number of cellular processes: proliferation and differentiation, DNA repair, and apoptosis. The biological function of circulating miRNAs is still under investigation. It has been suggested that circulating miRNAs may originate from cell destruction in the course of carcinogenesis. Other theories assume that miRNAs are released from the cell in a manner dependent on microvesicles (MVs), or actively and selectively secreted independently and free from MVs in response to a variety of stimuli [29]. Based on the analysis of specific miRNAs present in the blood, it is possible to determine what type of cells they come from [30]. MiRNAs in plasma, serum, freshly frozen tissues, paraffin blocks and saliva are resistant to endogenous and exogenous RNases, extreme temperatures, and pH. They are very stable for a long time, even if left at room temperature [31,32]. They are seen as potential biomarkers that can be obtained in a minimally invasive manner [33,34,35,36].

A number of miRNA molecules with potential prognostic value in MM were designated based on recent results. However, due to differences in the studied material, the results are inconsistent, and miRNAs are still not used in routine clinical practice. Xu et al. undertook a systematic review of the literature to verify the potential usefulness of miRNAs in the assessment of prognosis in patients with MM. In a meta-analysis of 10 studies and 1214 patient cases, they found that only seven miRNAs were assessed at least twice, with the majority being analyzed in a single study only. The results of the meta-analysis showed that a lower expression of miR-15a, miR-16, miR-25, miR-744 and let-7e was a predictor of worse OS in MM patients. On the other hand, a decrease in the level of miR-15a, miR-16 and miR-25 and an increase in the level of miR-92a were associated with a shorter PFS [37].

The best known miRNAs are miR-15a and miR-16. They are located on the chromosomal 13q14 site and have similar sequences. They perform tumor-suppressor functions and are involved in the regulation of cell proliferation, differentiation, and apoptosis. They are also involved in angiogenesis in many cancers, including MM [38,39,40,41,42]. Roccaro et al. demonstrated that miR-15a/16-1 regulates MM cell proliferation in vitro and in vivo by inhibiting the AKT serine/threonine protein kinase, ribosomal S6 protein, MAP kinases and the NF-kB MAP3KIP3 activator [43]. Other studies have shown that miR-15a/16-1 targets such genes as *BCL2, MCL1, CCND1, WNT3A* and *VEGF* [44]. MiR-15a/16 negatively regulates the expression of calcineurin binding protein 1 (CABIN1) in MM cells directly through mRNA. As a result, a decreased expression of miR-15a and miR-16 in MM promotes proliferation by increasing CABIN1 protein expression [45].

MiR-25 is transcribed as part of the mir-106b-25 polycistrone, having both oncogenic and suppressor functions. It is abnormally expressed in many cancers. The level of miR-25 is decreased in ovarian cancer [46], while it is increased in brain tumors in children, medulloblastomas, prostate cancer, hepatocellular carcinoma, gastric cancer, lung adenocarcinoma, colorectal cancer and in MM [47,48,49,50,51,52]. Rocci et al. assessed 10 selected miRNAs, including serum miR-25 in 288 patients with newly diagnosed MM. The authors showed that the level of circulating miR-25 was significantly associated with both PFS (*p* = 0.034) and OS (*p* = 0.0005), and a higher S-miR-25 expression was associated with a longer duration of PFS (HR = 0.92; *p* = 0.034) [53]. However, in another study, Zi et al. observed that an increased expression of miR-25-3p in patients with MM is associated with anemia and a higher disease stage in the ISS and Durie–Salmon classifications; therefore, a high level of miR-25-3p may predict poor prognosis in patients with MM. In in vitro studies, authors have demonstrated that miR-25-3p knockdown inhibits proliferation and promotes MM cell apoptosis via the PTEN/PI3K/AKT signaling pathway [54].

Kubiczkowa et al. were the first to report that the expression of miR-744 and let-7e in the serum of MM patients is reduced (*p* < 0.001), and that they can be used as markers in the assessment of prognosis. One-year mortality rates for miR-744 and let-7e were 41.9% and 34.6% for the “low” expression and 3.3% and 3.9% for the “high” expression groups, respectively. The median durations of remission for both miR-744 and let-7e in MM patients were approximately 11 months for “low” expression and 47 months for “high” expression [55]. In a recently published study, Guo et al. confirmed that the expression of miR-744-5p in bone marrow tissue obtained from patients with MM is reduced compared to healthy controls (*p* < 0.01). Low miR-744-5p expression was associated with worse 60-month survival (*p* = 0.04021). In vitro studies proved that miR-744-5p levels were significantly reduced in MM cell lines (NCI-H929, KM3, H929, U1996 and U266) compared to normal plasmocytes (nPC—normal Plasma Cells) (*p* < 0.01 or *p* < 0.001). The authors undertook the challenge of explaining the molecular mechanisms of miR-744 in the pathogenesis of MM. They showed that the overexpression of miR-744-5p inhibits proliferation, invasion, migration, glucose uptake, lactic acid production and epithelial mesenchymal transformation (*p* < 0.01) by targeting the SOX12/Wnt/β-catenin pathway [56].

It has been proved that MM cells are characterized by a strong expression of the miR-17-92a cluster [57]. A higher level of miR-92a expression on neoplastic plasmocytes is associated with poor prognosis. Qu et al. reported the median PFS in patients with high miR-92a expression as 4.5 months, while in patients with low expression it reached 14.0 months (*p* = 0.006) with a median follow-up of 13.5 (0.5–72.5) months. The authors suggested that miR-92a influences the course of MM via the c-jun pathway [58]. Yoshizawa et al. assessed circulating miR-92a in patients with various stages pf MM and in patients with related diseases. Patients with symptomatic MM had significantly reduced miR-92a expression (*p* < 0.0001) in plasma and in the CD8+ or CD4+ cells of peripheral blood compared to healthy controls. The levels of circulating miR-92a in the group of patients with complete remission (CR) were normalized, which was not observed in the group with a partial response (PR) and very good PR (VGPR). The authors suggested that measuring the plasma level of miR-92a in patients with MM could be a useful tool in qualifying patients for the initiation of chemotherapy and for monitoring disease status, and that it may partially represent the immune status of patients’ T cells [59].

In this study, we confirmed the prognostic value of well-known clinical factors (performance status, type of MM, the stage of disease, and kidney function) and laboratory biomarkers (hemoglobin, platelets, albumin, creatinine, calcium, LDH, β2-microglobulin, del(17p), and t(14;16)) in MM. Interestingly, we observed that the presence of t(11;14) was associated with a poorer clinical outcome. PFS was significantly shorter in patients with t(11;14) (median 8.0 vs. 28.0 months, *p* = 0.0033). t(11;14) is not currently considered a high-risk cytogenetic aberration in MM. However, since there is increasing evidence of shorter PFS and OS in patients with t(11;14), it might be reasonable to consider it as an intermediate risk marker [60,61,62]. Moreover, we analyzed the expression of circulating miRNA-8074 in MM patients, and we correlated the obtained results with detailed clinical data and treatment outcomes. 

So far, very limited data on the biological functions of miRNA-8074 are available. Francavilla et al. assessed the relationship between stool miRNA levels and age, sex, BMI, and lifestyle habits in healthy individuals, using small RNA-sequencing data of samples from 335 healthy subjects. The authors found that miRNA-8074 levels decreased with age (−0.26, adj. *p* <  0.001) [63]. Kolhe et al. noted that synovial fluid exosomal miRNA-8074 is down-regulated in females with osteoarthritis [64]. In another study, miRNA-8074 was upegulated in mesenchymal stem/stromal cell-derived extracellular vesicles in patients with metabolic syndrome [65]. Only two reports of miR-NA-8074 focused on cancers. An association between miRNA-8074 and the risk of breast cancer has not been confirmed [66]. In another study, Xu et al. explored 2549 exosomal miRNAs in lung adenocarcinoma, and found that miRNA-8074 was one of ten molecules that were upregulated [67].

To the best of our knowledge, the role of miRNA-8074 in MM pathogenesis has not been studied before. miRNA-8074 is hypothetically able to regulate several key signaling pathways involved in the pathogenesis of MM, which has piqued our scientific interest. In our study, we did not observe significant differences or correlations between miRNA-8074 expression and any of the selected demographic, clinical and molecular variables. We found no difference in expression levels depending on the response to chemotherapy. However, serum miRNA showed a positive correlation with intracellular levels in malignant CD138+ bone marrow plasma cells in paired samples. We noticed that a high miRNA-8074 expression was associated with a higher risk of PFS shortening (17 vs. 39 months; HR = 2.01, 95% CI: 1.16–3.49; *p* = 0.0233) and a higher risk of OS shortening (30 months vs. NR; HR = 4.12, 95% CI: 2.20–7.70; *p* = 0.0009). We confirmed the obtained results in a multivariate analysis. A high expression of miRNA-8074 was associated with a shorter PFS (17 vs. 39 months; HR = 2.67, 95% CI: 1.15–6.20, *p* = 0.0231) and OS (30 months vs. NR; HR = 5.25, 95% CI: 1.38–19.97; *p* = 0.0155). A positive correlation between miRNA-8074 and *MYC* gene expression (rho = 0.476) could partially explain the role of miRNA-8074 in MM biology.

Thus, we demonstrated that serum miRNA-8074 expression has the potential to be a simple, non-invasive prognostic biomarker in patients with MM. The weak points of our project are the limited functional studies that would explain the molecular mechanism of action of miRNA-8074 in the pathogenesis and development of MM. Another limitation is the relatively small number of patients treated with various first-line treatment regimens. However, this is the first pilot study to explore the clinical relevance of miRNA-8074 in MM. Our observations that serum miRNA-8074 expression could be used as a novel prognostic marker for MM indicate the need for additional research in this direction.

## 5. Conclusions

Understanding the profile of circulating miRNAs can provide a lot of valuable information related to the diagnosis of MM and allow for further risk stratification. So far, several circulating miRNAs with predictive and prognostic potential in MM have been identified. Although our results are preliminary, they indicate that the expression of circulating miRNA-8074 may be useful in patients with MM.

## Figures and Tables

**Table 1:** Baseline characteristics of the study group.

Variable	n = 105 (100%)
**Sex**	
Men	52 (49.5%)
Women	53 (50.6%)
**Age**	
<65	35 (33.3%)
≥65	70 (66.7%)
**Diagnosis**	
MM with a monoclonal component	88 (83.8%)
Light chain disease	16 (15.2%)
Non-secretory MM	1 (0.9)
**Monoclonal protein class**	
IgA	29 (27.6%)
IgG	59 (56.2%)
**Light chain type**	
Kappa	64 (61.5%)
Lambda	40 (38.5%)
**Durie–Salmon stage**	
I	9 (8.6%)
II	14 (13.3%)
III	82 (78.1%)
**ISS stage**	
1	29 (27.9%)
2	30 (28.8%)
3	45 (43.3%)
No data: n = 1	
**Renal function**	
A-creatinine < 2 mg/dL	87 (82.9%)
B-creatinine ≥ 2 mg/dL	18 (17.1%)
**Performance status**	
0	8 (7.9%)
1	43 (40.9%)
2	42 (40.0%)
3	10 (9.9%)
4	2 (2.0%)
**Body weight loss**	
No	42 (49.4%)
Yes	43 (50.6%)
No data: n = 20	
5%	14 (32.6%)
10%	29 (67.4%)
**Anemia grade (WHO)**	
Absent or I	17 (16.2%)
II, III or IV	88 (83.8%)
**Treatment protocol**	
CTD	27 (25.7%)
V(C)D	41 (39.0%)
VTD	37 (35.2%)
**del(17p13.1)**	
Absent	74 (87.1%)
Present	11 (12.9%)
No data: n = 20	
**t(4;14)**	
Absent	75 (88.2%)
Present	10 (11.8%)
No data: n = 20	
**t(14;16)**	
Absent	84 (98.8%)
Present	1 (1.2%)
No data: n = 20	
**t(11;14)**	76 (89.4%)
Absent	9 (10.6%)
Present	
No data: n = 20	
**Other *IgH* gene rearrangement**	
Absent	72 (84.7%)
Present	13 (15.3%)
No data: n = 20	

CTD—cyclophosphamide, thalidomide, dexamethasone; ISS—Multiple Myeloma International Staging System; WHO—World Health Organization; V(C)D—bortezomib, (cyclophosphamide), dexamethasone; VTD—bortezomib, thalidomide, dexamethasone.

**Table 2:** Median PFS and OS of MM patients depending on selected factors.

Variable	Progression-Free Survival			Overall Survival		
	Univariate		Multivariate ^c^	Univariate		Multivariate ^d^
	*Me* (months)	*p*	*p*	*Me* (months)	*p*	*p*
	16	HR (95% CI)	HR (95% CI)	24	HR (95% CI)	HR (95% CI)
**Sex**						
Men	24	0.0603	0.1202	NR	0.1665	0.1213
Women	37	1.65 (0.97–2.80)	1.83 (0.86–3.92)	31	1.52 (0.84–2.77)	2.04 (0.83–4.98)
**Age**						
<65	39	0.0929	0.0622	33	0.8347	0.3608
≥65	17	0.60 (0.35–1.05)	0.42 (0.17–1.04)	41	1.07 (0.57–2.01)	1.64 (0.57–4.69)
**Diagnosis**						
Light chain disease, Non-secretory MM	15	0.0987	0.5534	24	0.0004 *	0.0928
MM with a monoclonal component	26	1.72 (0.78–3.80)	1.47(0.41–5.25)	NR	3.01 (1.22–7.42)	2.77 (0.85–9.06)
**Monoclonal protein class**						
IgA	26	0.8713	0.13	NR	0.2565	0.3174
IgG	25	1.05 (0.56–1.97)	2.11 (0.81–5.54)	NR	1.50 (0.69–3.23)	1.73 (0.60–5.00)
**Light chain type**						
Lambda	28	0.2866	0.7216	41	0.2857	0.992
Kappa	17	0.75 (0.43–1.32)	0.87 (0.41–1.86)	33	0.72 (0.38–1.37)	0.99 (0.39–2.54)
**Durie Salmon stage**						
III	17	0.9544	0.0833	31	0.0101 *	0.0014 *
I, II	25	1.02 (0.53–1.94)	2.21 (0.90–5.40)	41	3.06 (1.31–7.15)	4.84 (1.84–12.72)
**ISS stage**						
3	17	0.0235 *	0.0116 *	33	0.1567	0.6298
1, 2	39	1.82 (1.05–3.15)	2.59 (1.24–5.41)	41	1.53 (0.83–2.83)	1.25 (0.51–3.06)
**Renal function**						
B	15	0.0044 *	0.8629	25	0.0354 *	0.6035
A	30	2.31 (1.05–5.10)	1.09 (0.40–3.01)	41	2.03 (0.87–4.76)	1.39 (0.40–4.86)
**Stage of chronic kidney disease**						
G3a/G3b/G4/G5D	7	0.0380 *	0.6402	41	0.4027	0.8098
G1/G2	25	2.50 (0.63–9.91)	1.07 (0.81–1.40)	NR	1.63 (0.38–6.99)	1.03 (0.78–1.37)
**Performance status**						
2–4	17	0.0478 *	0.2741	29	0.0023 *	0.4613
0, 1	42	1.73 (1.00–2.98)	1.51 (0.72–3.17)	NR	2.64 (1.42–4.88)	1.40 (0.57–3.42)
**Treatment protocol (1)**						
CTD	24	0.2633	0.2464	33	0.9705	0.9167
V(C)D, VTD	25	1.35 (0.76–2.40)	1.83 (0.66–5.07)	33	1.01 (0.53–1.92)	1.07 (0.31–3.63)
**Treatment protocol (2)**						
CTD, V(C)D	17	0.0033 *	0.0168 *	33	0.3191	0.6251
VTD	NR	2.64 (1.53–4.57)	2.75 (1.21–6.28)	41	1.41 (0.74–2.66)	1.28 (0.48–3.43)
**Body weight loss before treatment**						
Yes	18	0.0816	0.3129	30	0.0763	0.3183
No	39	1.67 (0.93–3.01)	1.60 (0.64–3.96)	NR	1.80 (0.93–3.46)	1.68 (0.61–4.63)
**Anemia grade before treatment (WHO)**						
Absent or I	39	0.1645	0.5548	41	0.9537	0.3495
II, III or IV	24	0.58 (0.30–1.13)	0.69 (0.20–2.39)	41	0.98 (0.47–2.03)	0.37 (0.05–2.93)
**Hemoglobin**						
Low	25	0.0459 *	0.5228	41	0.8786	0.2902
Normal	NR	2.42 (1.24–4.72)	1.52 (0.42–5.52)	33	1.06 (0.48–2.32)	1.89 (0.58–6.12)
**Platelets**						
Low	17	0.0233 *	0.0303 *	33	0.8238	0.4022
Normal	39	1.81 (1.04–3.15)	2.54 (1.10–5.90)	41	1.07 (0.58–1.98)	1.57 (0.55–4.49)
**Albumins**						
Low	13	0.0002 *	0.0280 *	25	0.0042 *	0.0899
Normal	39	2.57 (1.45–4.57)	2.35 (1.10–5.03)	NR	2.29 (1.21–4.38)	2.15 (0.89–5.18)
**CRP**						
High	24	0.5561	0.299	31	0.1484	0.29
Normal	26	1.17 (0.67–2.06)	1.53 (0.69–3.43)	NR	1.56 (0.81–3.01)	1.61 (0.67–3.85)
**LDH**						
High	7	0.0041 *	0.0173 *	15	0.0007 *	0.0018 *
Normal	25	2.58 (0.97–6.87)	3.28 (1.24–8.66)	NR	3.01 (1.06–10.30)	5.94 (1.95–18.11)
**Calcium**						
High	24	0.293	0.3505	28	0.0386 *	0.1036
Normal	25	1.35 (0.73–2.49)	0.64 (0.26–1.61)	NR	1.88 (0.93–3.80)	2.08 (0.86–5.00)
**B2M**						
High	24	0.0552	0.8337	41	0.0066 *	0.5968
Normal	NR	2.89 (1.35–6.17)	1.16 (0.28–4.76)	41	3.12 (1.38–7.05)	0.65 (0.13–3.22)
**Creatinine**						
High	14	0.0003 *	0.0699	28	0.0622	0.3451
Normal	39	2.54 (1.742–4.54)	2.31 (0.94–5.66)	NR	1.74 (0.92–3.29)	1.74 (0.56–5.42)
**eGFR**						
Low	15	0.0051 *	0.7065	28	0.0623	0.8429
Normal	39	2.08 (1.17–3.70)	1.21 (0.45–3.26)	NR	1.75 (0.92–3.31)	1.13 (0.33–3.95)
**del(17p)**						
Present	15	0.0410 *	0.7902	30	0.068	0.0816
Absent	28	2.08 (0.81–5.37)	1.14 (0.44–2.96)	NR	2.21 (0.68–7.23)	2.85 (0.88–9.22)
**t(4;14)**						
Present	NR	0.343	0.6425	NR	0.8738	0.7077
Absent	24	1.74 (0.68–4.43)	1.38 (0.35–5.44)	41	1.10 (0.35–3.46)	1.30(0.34–4.99)
**t(14;16)**						
Present	9	0.1089	0.1191	2	<0.0001 *	0.0012 *
Absent	25	4.29 (0.07–242.17)	7.15 (0.61–83.86)	41	21.92 (0.00–18528.31)	81.76 (5.81–11,150.18)
**t(11;14)**						
Present	8	0.0033 *	0.0003 *		0.7822	0.4937
Absent	28	2.94 (0.93–9.33)	5.66 (2.22–14.42)	31	1.16 (0.38–3.51)	1.55 (0.44–5.45)
**Other *IgH* gene rearrangement**						
Present	25	0.9699	0.9648	NR	0.3936	0.8497
Absent	25	1.01 (0.45–2.27)	0.98 (0.37–2.61)	41	1.66 (0.62–4.44)	0.88 (0.24–3.18)
**miRNA-8074 expression (according to the median value)**						
High	17	0.0233 *	0.0343 *	30	0.0009 *	0.0142 *
Low	39	2.01 (1.16–3.49)	2.28 (1.07–4.91)	NR	4.12 (2.20–7.70)	3.97 (1.32–11.90)

B2M—beta-2-microglobulin; CI—confidence interval; CRP—C-reactive protein; eGFR—estimated glomerular filtration rate; HR—hazard ratio; ISS—Multiple Myeloma International Staging System; LDH—lactate dehydrogenase; *Me*—median; NR—not reached; *p*—statistical significance; WHO—World Health Organization. ^c^—results adjusted for variables demonstrating statistical significance in a univariate analysis of PFS. ^d^—results adjusted for variables demonstrating statistical significance in a univariate analysis of OS, *—statistically significant result.

## Data Availability

The data presented in this study are available from the corresponding author upon reasonable request. They are not publicly available due to the fact that the data sheet contains information that exceeds the scope of this study and which may be used for other research papers in the future.

## Supplementary Materials

The following are available online at https://www.mdpi.com/article/10.3390/cells11040752/s1, Figure S1: Scatter plots representing correlations between the expression of miRNA-8074, TP53 gene [A] and MYC gene [B] in bone marrow plasma cells as well as with the expression of miRNA-8074 in blood serum [C]. Table S1: Comparison of the miR-8074 expression depending on demographic, clinical and molecular factors. Table S2: Spearman‘s rank correlation between selected demographic, clinical and molecular variables and miR-8074 expression. Table S3: Comparison and assessment of the usefulness of the miRNA-8074 expression level in differentiating CTH responses.

## Abbreviations

CTCAE—Common Terminology Criteria for Adverse Events; CTD—cyclophosphamide, thalidomide, dexamethasone; FISH—fluorescence in situ hybridization; HR—hazard ratio; IMiD—immunomodulatory drug; IMWG—International Myeloma Working Group; ISS—Multiple Myeloma International Staging System; MM—multiple myeloma; PFS—progression-free survival; PI—proteasome inhibitor; V(C)D—bortezomib, (cyclophosphamide), dexamethasone; WHO—World Health Organization; VTD—bortezomib, thalidomide, dexamethasone.
