# Supplementary material for: Circulating Serum MiRNA-8074 as a Novel Prognostic Biomarker for Multiple Myeloma
Source: Cells. 2022 Feb 21;11(4):752. doi: 10.3390/cells11040752 (PMC8870602; doi:10.3390/cells11040752)
Supplement: Supplementary file 1 [file cells-11-00752-s001.zip › cells-1526915-supplementary.pdf]

Supplementary materials

Table S1. Comparison of the *miR-8074* expression depending on demographic, clinical and molecular factors.

Variable	*miR-8074*	*P*
	Me	
	0.88	
**Sex**		
**Men**	0.69	0.4788
**Women**	2.02	
**Age**		
<65	2.08	0.8677
≥65	0.79	
**Diagnosis**		
MM with a monoclonal component	1.42	0.7774
Light chain disease	0.54	
**Monoclonal protein class**		
IgA	3.11	0.3803
IgG	0.79	
**Light chain type**		
Kappa	0.63	0.2926
Lambda	2.13	
No data: n=1		
**Durie-Salmon stage**		
I	2.80	
II	2.36	0.4108
III	0.57	
**ISS stage**		
1	0.49	
2	1.80	0.8241
3	1.26	
No data: n=1		
**Renal function**		
A - creatinine < 2 mg/dL	1.59	0.7306
B - creatinine ≥ 2 mg/dL	0.55	
**Performance status**		
**0**	0.39	
**1**	0.41	
**2**	1.95	0.4151
**3**	4.36	
**4**	6.26	
**Body weight loss**		
No	0.46	0.3425
Yes	1.78	
No data: n=20		
5%	2.06	0.3121
10%	0.59	
**Anemia grade (WHO)**		
Absent or I°	0.26	0.2266
II°, III° or IV°	1.60	
**Treatment protocol**	1.61	0.5759
CTD	0.72	

V(C)D<br>VTD	1.88	
**del (17p)**<br>Absent<br>Present<br>*No data: n=20*	0.84<br>0.41	0.5085
**t(4;14)**<br>Absent<br>Present<br>*No data: n=20*	0.81<br>0.52	0.9674
**t(14;16)**<br>Absent<br>Present<br>*No data: n=20*	0.75<br>1.61	-
**t(11;14)**<br>Absent<br>Present<br>*No data: n=20*	1.42<br>0.20	0.2971
**Other *IgH* gene rearrangement**<br>Absent<br>Present<br>*No data: n=20*	0.64<br>1.88	0.9173

CTD - cyclophosphamide, thalidomide, dexamethasone; ISS - Multiple Myeloma International Staging System; *Me* – median, *p* - statistical significance, WHO - World Health Organization; V(C)D - bortezomib, (cyclophosphamide), dexamethasone; VTD - bortezomib, thalidomide, dexamethasone.

Table S2. Spearman`s rank correlation between selected demographic, clinical and molecular variables and *miR-8074* expression.

Variable	***miR-8074* expression**	
	**rho**	***P***
**Age [years]**	0.0421	0.6701
**Serum monoclonal protein (g/dl)**	0.112	0.6286
**Serum light chain (mg/l)**	0.161	0.4743
**Durie-Salmon stage**	-0.128	0.1940
**ISS stage**	0.0260	0.7930
**Kidney disease A/B**	-0.121	0.2190
**Performance status**	0.189	0.0583
**BMI**	0.0172	0.8852
**Weight loss [%]**	0.104	0.3455
**Haemoglobin [g/dl]**	-0.114	0.2483
**Anaemia grade (WHO)**	0.119	0.2283
**Lymphocytes [K/ul]**	0.0753	0.4452
**Neutrophils [K/ul]**	-0.0319	0.7469
**Neutrophil to lymphocyte ratio**	-0.0694	0.4839
**Platelets [K/ul]**	0.0557	0.5722
**MPV [fl]**	-0.102	0.2992
**Number of lytic foci in the bones**	-0.0757	0.4449
**eGFR [mL/min/1.73m2]**	0.0838	0.3974
**Stage of chronic kidney disease**	-0.121	0.2190
**Albumins [mg/dl]**	-0.159	0.1045

**Creatinine [mg/dl]**	-0.0860	0.3832
**B2M [ng/l]**	0.0502	0.6144
**LDH [IU/l]**	-0.0389	0.7049
**Calcium [mmol/l]**	-0.0172	0.8619
**CRP [mg/l]**	0.0352	0.7231
**del (17p) [% cell.]**	-0.0721	0.4169
**t(4;14) [% cell.]**	-0.00447	0.9537
**t(14;16) [% cell.]**	-0.114	0.8235
**t(11;14) [% cell.]**	-0.114	0.2018
**Other *IgH* gene rearrangement [% cell.]**	-0.0113	0.8937

auto HSCT - autologous hematopoietic stem cell transplant, B2M – beta-2-microglobulin, , CRP – C-reactive protein, eGFR – estimated glomerular filtration rate, ISS - Multiple Myeloma International Staging System, LDH - lactate dehydrogenase, MPV – mean platelet volume, *p* - statistical significance, rho- Spearman's rank correlation coefficient, WHO - World Health Organization.

Table S3. Comparison and assessment of the usefulness of the *miRNA-8074* expression level in differentiating CTH responses.

**Variable**	***miRNA-8074*expression**						
	Me	***p*[a]**	**cut-off**	**Sensitivity**	**Specificity**	**AUC (95%CI)**	***p*[b]**
**Response after 2 cycle of CTH**<br>No<br>Yes	**0.79**<br>**3.08**	0.5511	**>0.539**	71.43%	48.84%	0.550 (0.45-0.65)	0.5229
**Response after 4 cycle of CTH**<br>No<br>Yes	**0.66**<br>**1.26**	0.3918	**>0.539**	85.71%	50.00%	0.598 (0.49-0.70)	0.2285
**Response after 6 cycle of CTH**<br>No<br>Yes	**0.79**<br>**0.56**	0.5568	**>0.108**	100.00%	35.53%	0.572 (0.46-0.68)	0.5172
**Response after 8 cycle of CTH**<br>No<br>Yes	**1.61**<br>**0.72**	0.7359	**>0.078**	100.00%	33.33%	0.548 (0.36-0.72)	0.7149

AUC – area under curve, CI – confidence interval, CTH – chemotherapy, *Me* -median, *p* - statistical significance.

Response: CR= complete response, VGPR = very god partial response, PR = partial response, MR = minimal response; Lack of response: SD = stable disease, PD - progressive disease.

Response criteria adapted from Durie et al (2006) [16].

[a] – *p* value for U-Mann-Whitney test, [b]– *p* value for ROC analysis.

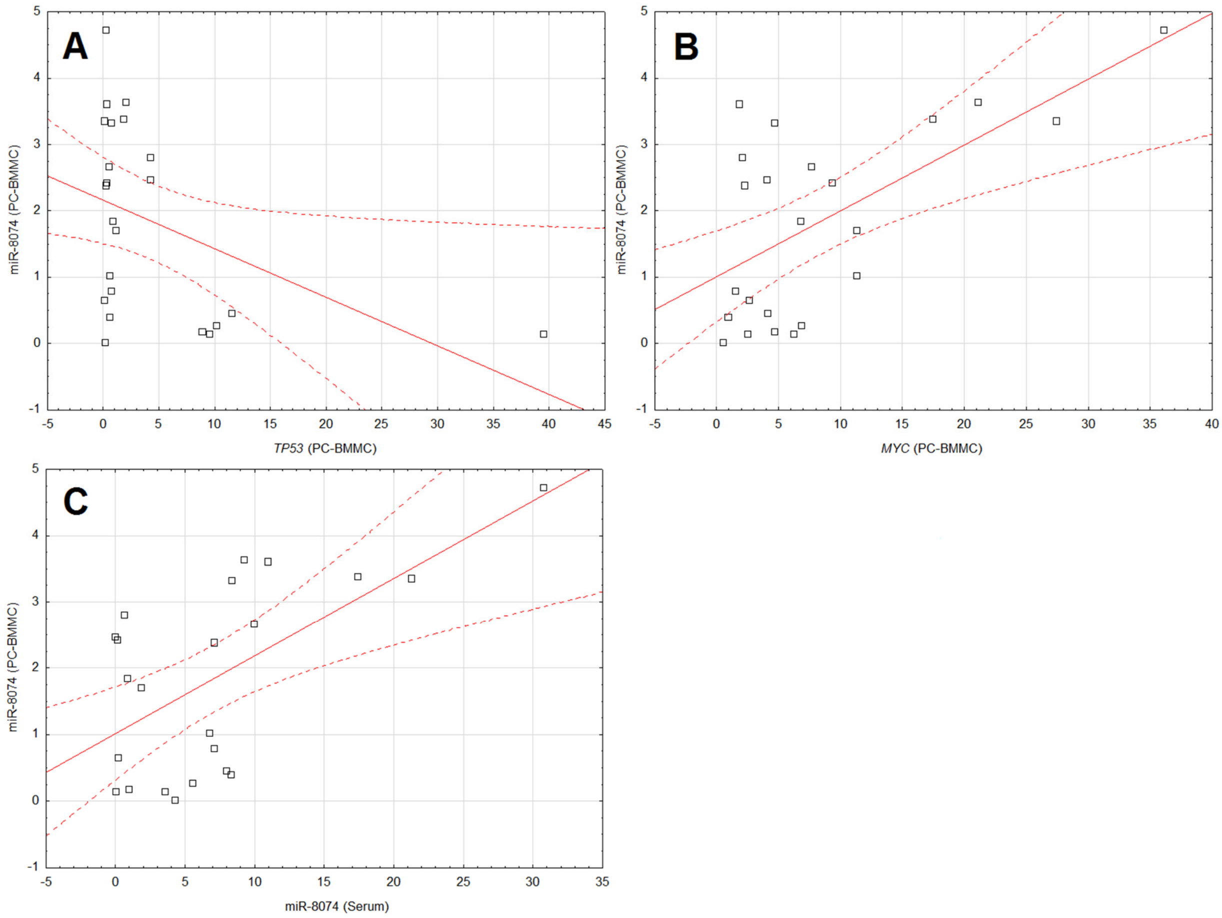

Figure S1. Scatter plots representing correlations between the expression of miRNA-8074, *TP53* gene [A] and MYC gene [B] in bone marrow plasma cells as well as with the expression of miRNA-8074 in blood serum [C].
